# 

**Published:** 1914-10-31

**Authors:** 


### Buildings Contemplated and in Progress.

Abergavenny.—The Guardians have decided that local architects be invited to send in competitive plans for a new workhouse, to cost £20,000.

Billericay.—A scheme has been prepared for structural alterations at the workhouse, to cost £4,827. The architect, Mr. H. R. Bird, has been instructed to confer with the architect of the Local Government Board on the matter.

Essex.—The Committee of Visitors invite tenders for an extension of the asylum, Goodmayes, Essex. Quantities and further particulars may be obtained at the office of the borough engineer, Town Hall, West Ham, E.

Leicester.—The Local Government Board have agreed to make a grant of £6,381 towards the cost, estimated at £10,636, of new sanatorium buildings adjoining the isolation hospital.

### How to Organise Alexandra Day.

### A LESSON IN PRICES FROM CARDIFF.

Although Alexandra Day was celebrated in Cardiff so long ago as June 25, the final result was only made known last week, when the Distribution Committee met under the presidency of the Lord Mayor of Cardiff, Dr. James Robinson. It was considered inexpedient to hold the meeting earlier, owing to the pressing claims of various organisations dealing with problems created by the war. The result has exceeded anticipations, revealing a balance of £1,650, which was allotted as follows: £1,250 to King Edward VII.'s Hospital, Cardiff; £125 each to the Royal *Hamadryad* Seamen's Hospital and the Cardiff Poor Cripples' Aid Society; and £50 each to three other organisations.

While the detailed results are of local interest, it may be noted for general information that the sale of Alexandra roses was on this occasion supplemented by an exceptionally well organised carnival, which yielded £614 of the total net result. A useful analysis of the receipts was supplied by the honorary auditor, Mr. Gilbert Shepherd, F.C.A., who pointed out that in most of the districts the amount collected in the boxes considerably exceeded the nominal selling price of the flowers sold, but that in other districts the contents of the boxes showed that the flowers had been sold at less than their nominal selling value. It was decided that next year the fixed price should generally be insisted upon as a minimum. Many tributes were paid during the meeting to the ladies and gentlemen whose organisation had proved so fruitful of success, and the Lady Mayoress and Mrs. Price Williams, the honorary secretary, were specially thanked. The latter was nominated for a presidency of King Edward VII.'s Hospital, Cardiff. Alexandra Day 1913 resulted in £1,060—£955 for the hospital.

### CONTENTS.

**Rates of Subscription:** Twelve months, 6/6; Six months, 4/-; Three months, 2/-, post free to any address in the United Kingdom. Abroad, Twelve months, 8/8; Six months, 5/-; Three months, 2/6, post free.—Address The Subscription Dept., "The Hospital," The Hospital Building, 28/29 Southampton Street, Strand, London W.C.

